# Supplementary material for: Structural basis for ligand recognition and signaling of hydroxy-carboxylic acid receptor 2
Source: Nat Commun. 2023 Nov 6;14:7150. doi: 10.1038/s41467-023-42764-8 (PMC10628104; doi:10.1038/s41467-023-42764-8)
Supplement: Supplementary file 1 — Supplementary Information [file 41467_2023_42764_MOESM1_ESM.pdf]

SUPPLEMENTARY MATERIALS

Structural basis for ligand recognition and signaling of hydroxy-carboxylic acid receptor 2

Jae-Hyun Park[1†], Kouki Kawakami[2†], Naito Ishimoto[1], Tatsuya Ikuta[2], Mio Ohki[1], Toru Ekimoto[3], Mitsunori Ikeguchi[3,4], Dong-Sun Lee[5], Young-Ho Lee[6,7,8,9,10,11], Jeremy R.H. Tame[1], Asuka Inoue[2]*, Sam-Yong Park[1]*

[1]Drug Design Laboratory, Graduate School of Medical Life Science, Yokohama City University, Tsurumi, Yokohama 230-0045, Japan.

[2]Graduate School of Pharmaceutical Sciences, Tohoku University, Sendai 980-8578, Japan.

[3]Computational Life Science Laboratory, Graduate School of Medical Life Science, Yokohama City University, Yokohama City University, Tsurumi, Yokohama 230-0045, Japan.

[4]HPC- and AI-driven Drug Development Platform Division, Center for Computational Science, RIKEN, Yokohama 230-0045, Japan.

[5]Bio-Health Materials Core-Facility Center and Interdisciplinary Graduate Program in Advanced Convergence Technology and Science, Jeju National University, Jeju 63243, Republic of Korea.

[6]Department of Systems Biotechnology, Chung-Ang University, Gyeonggi 17546, Republic of Korea.

[7]Frontier Research Institute for Interdisciplinary Sciences, Tohoku University, Miyagi 980-8578, Japan

[8]Research Center for Bioconvergence Analysis, Korea Basic Science Institute, Ochang, Chungbuk 28119, Republic of Korea.

[9]Bio-Analytical Science, University of Science and Technology, Daejeon 34113, Republic of Korea.

[10]Graduate School of Analytical Science and Technology, Chungnam National University, Daejeon 34134, Republic of Korea.

[11]Research Headquarters, Korea Brain Research Institute, Daegu 41068, Republic of Korea.

†These authors contributed equally to this work.
*Corresponding author.
Email: iaska@tohoku.ac.jp (A.I.); park@yokohama-cu.ac.jp (S-Y.P.)

Supplementary Figure 1.

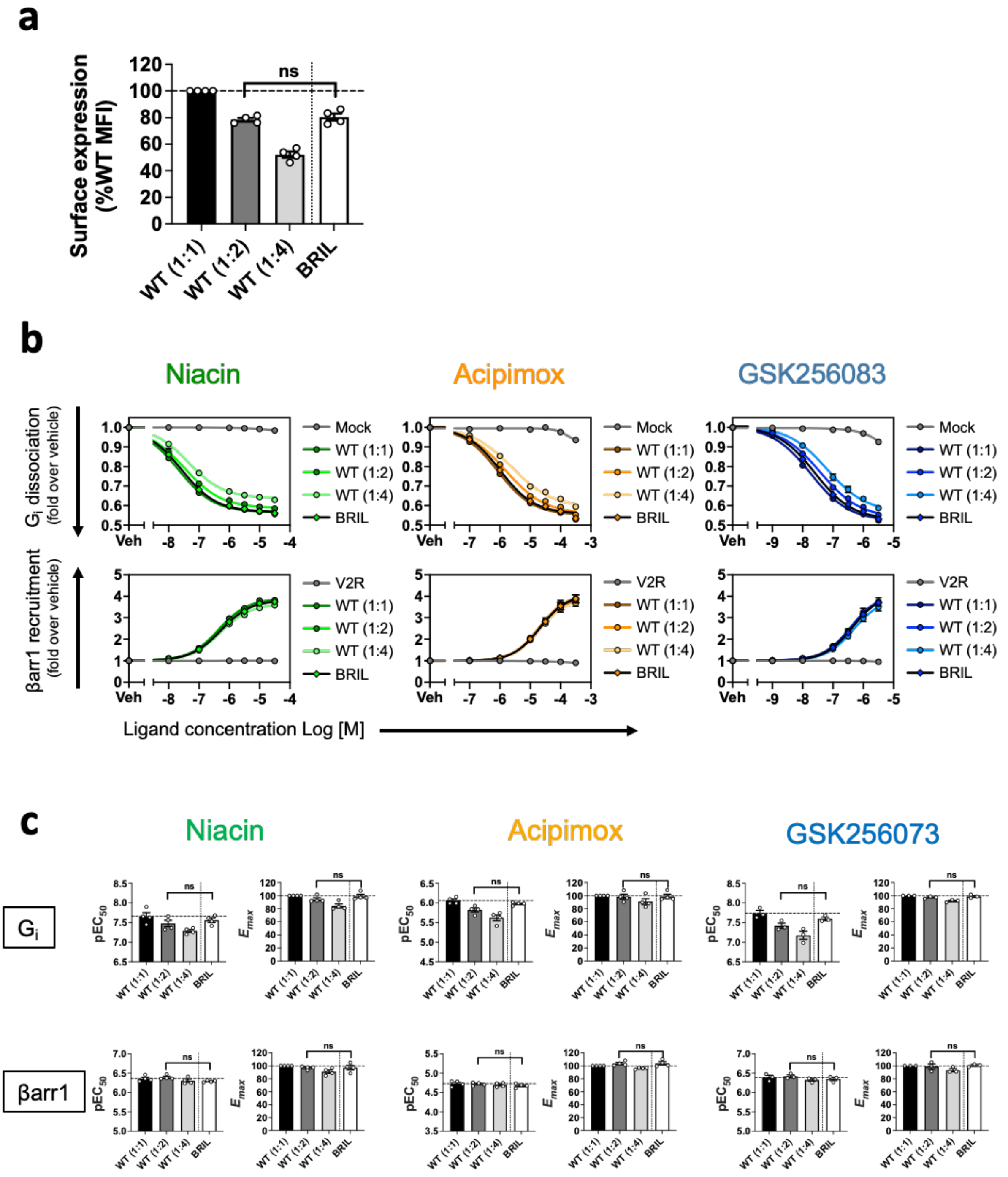

Supplementary Figure 1. Characterization of the BRIL-fused HCAR2 construct. (a) Surface expression levels of the wild-type and the BRIL-fused HCAR2. 1:1, 1:2 and 1:4 denote volume of transfected plasmids. MFI, mean fluorescent intensity. (b, c) The NanoBiT assays for G_i dissociation and β-arrestin1 (βarr1) recruitment upon stimulation with niacin, acipimox and GSK256073. Concentration-response curves (b) and pharmacological parameters (c) are indicated. As a negative control for the NanoBiT-β-arrestin1 assay, the V2R-SmBiT construct was used instead of the HCAR2-SmBiT construct. Bars and error bars represent mean and SEM, respectively, of four (a) or three (b) independent experiments with each data point represented as a dot. ns, not significantly different between the groups.

Supplementary Figure 2.

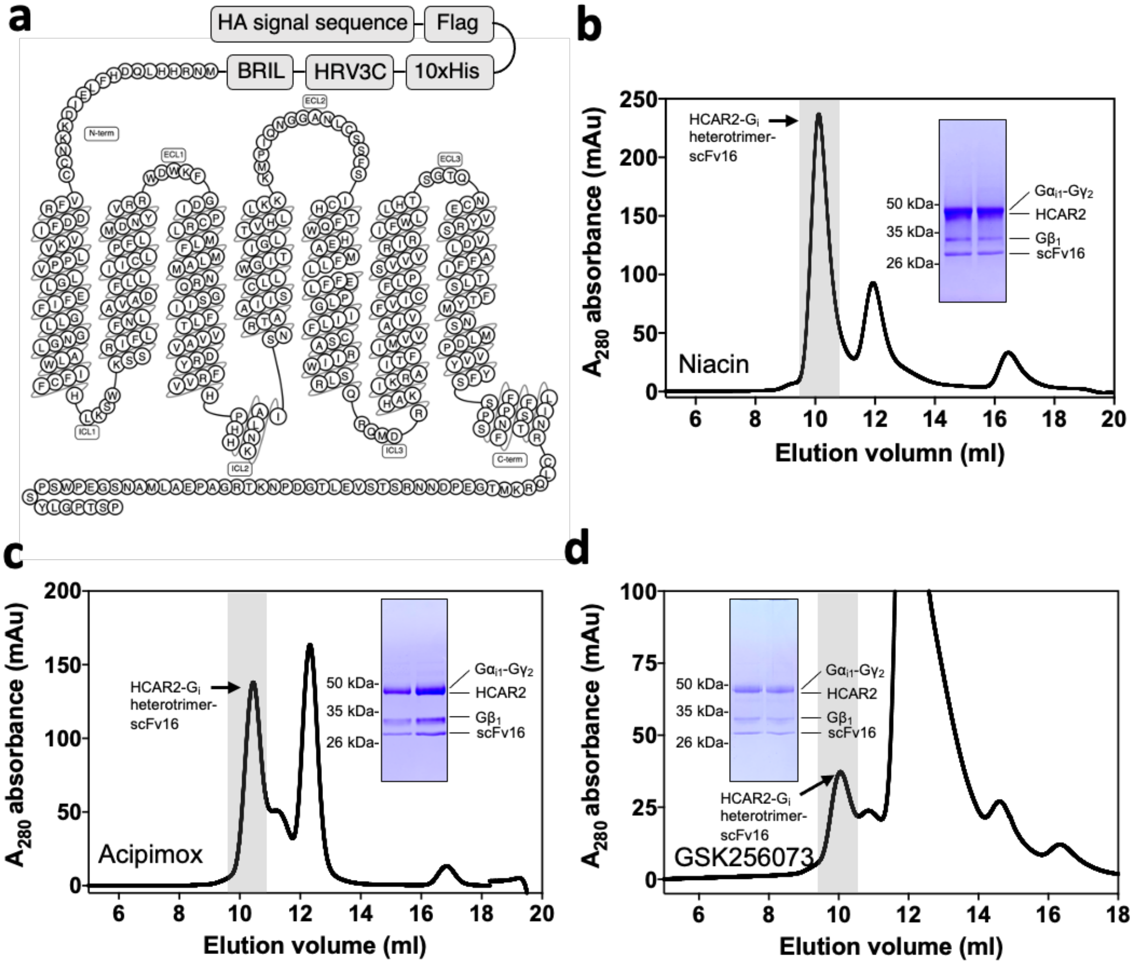

Supplementary Figure 2. Construction and purification of HCAR2-G$_i$ complexes. (a) Snake plot of the HCAR2 construct used for structural studies. Representative size-exclusion chromatography profiles of (b) Niain-HCAR2-G$_i$, (c) Acipimox-HCAR2-G$_i$, and (d) GSK256073-HCAR2-G$_i$ complexes. Insets show SDS–PAGE gels of the HCAR2-G$_i$ complex samples used for cryo-EM grid preparation, stained with Coomassie-blue.

Supplementary Figure 3.

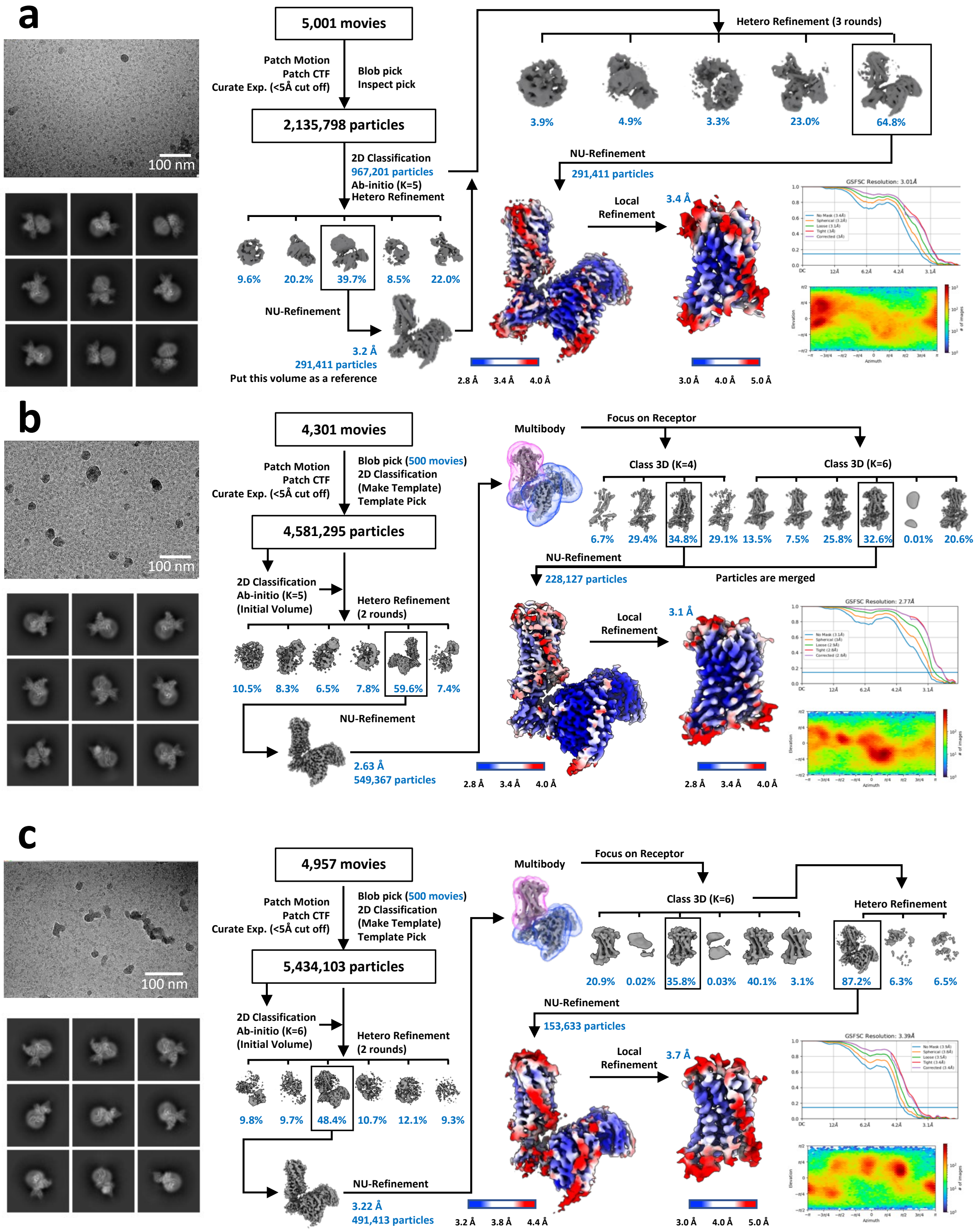

Supplementary Figure 3. Cryo-EM data collection and processing. Cryo-EM data processing workflow for HCAR2-G_i in presence of (a) niacin, (b) acipimox and (c) GSK256073. Representative micrographs and 2D classes of the complexes are shown. Gold-standard FSC curves indicate overall nominal resolutions using the FSC = 0.143 criterion.

Supplementary Figure 4.

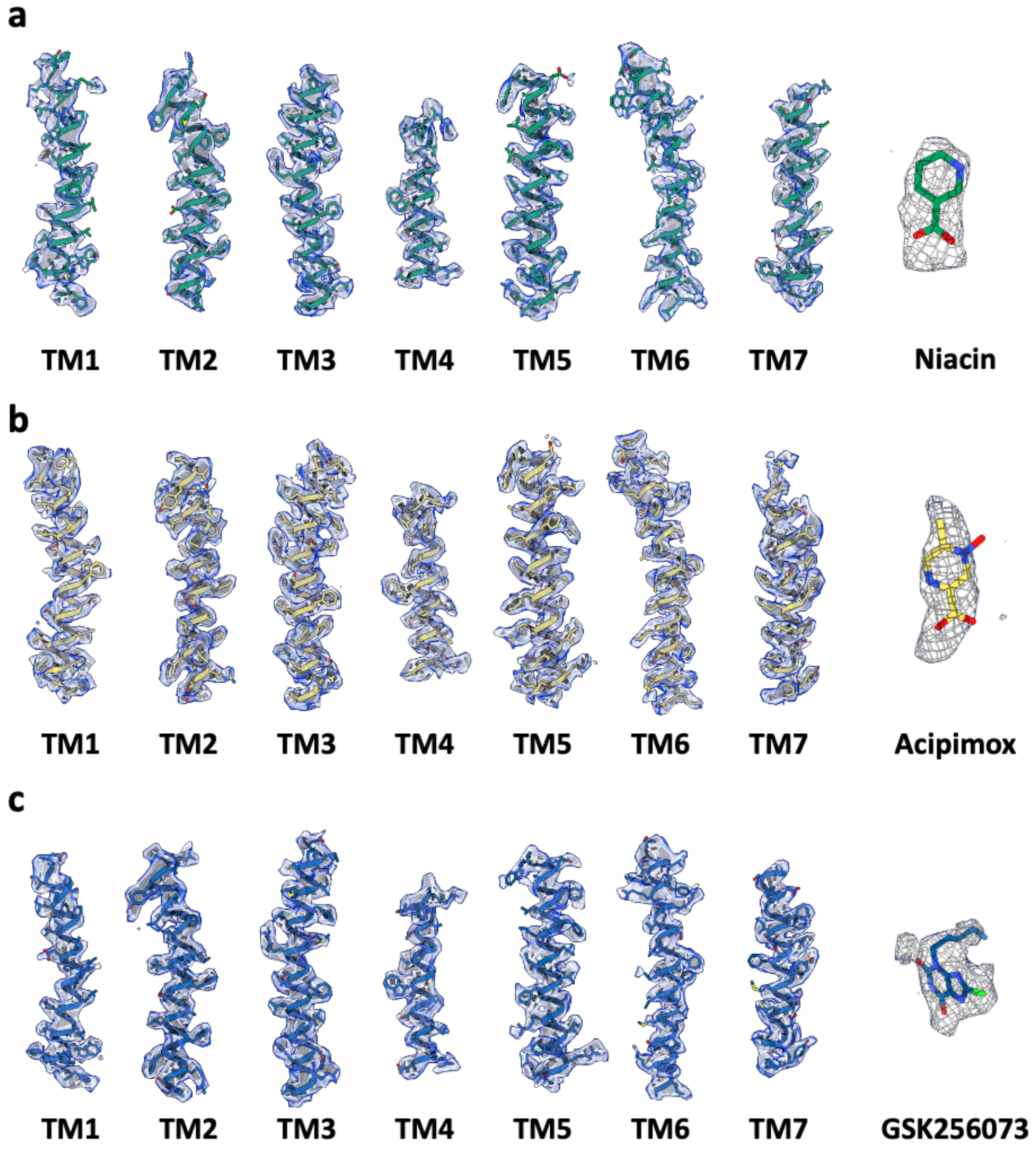

Supplementary Figure 4. Cryo-EM density maps of the refined structures of HCAR2 complexes. The cryo-EM density maps covering transmembrane helices of the HCAR2 complexes with (a) niacin, (b) acipimox and (c) GSK256073. Level of the maps was set to 0.25 in ChimeraX. The maps are displayed to show distances up to 2 Å around the model.

Supplementary Figure 5.

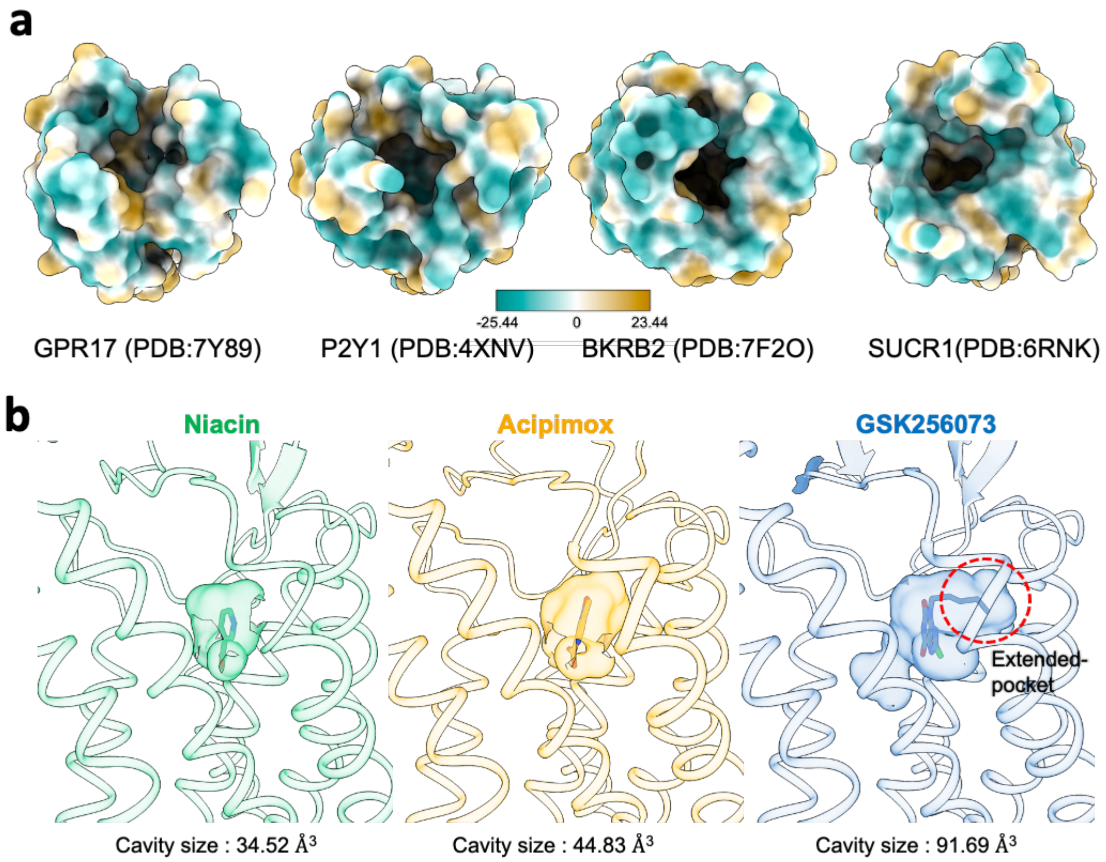

Supplementary Figure 5. Molecular surface of other GPCRs and ligand binding pocket of HCAR2.
Molecular surfaces of (a) GPR17 (PDB:7Y89), P2Y purinoceptor 1 (P2Y1; PDB:4XNV), Bradykinin Receptor B2 (BKRB2; PDB:7F2O) and Succinate Receptor 1 (SUCR1; PDB:6RNK). Hydrophobic and hydrophilic surface areas are colored yellow and blue, respectively (b) Structural models showing the pocket occupied by niacin, acipimox and GSK256073. Pocket volumes were calculated using CASTp 3.0. The extended ligand pocket of the GSK256073 bound structure is highlighted by a red dashed line.

Supplementary Figure 6.

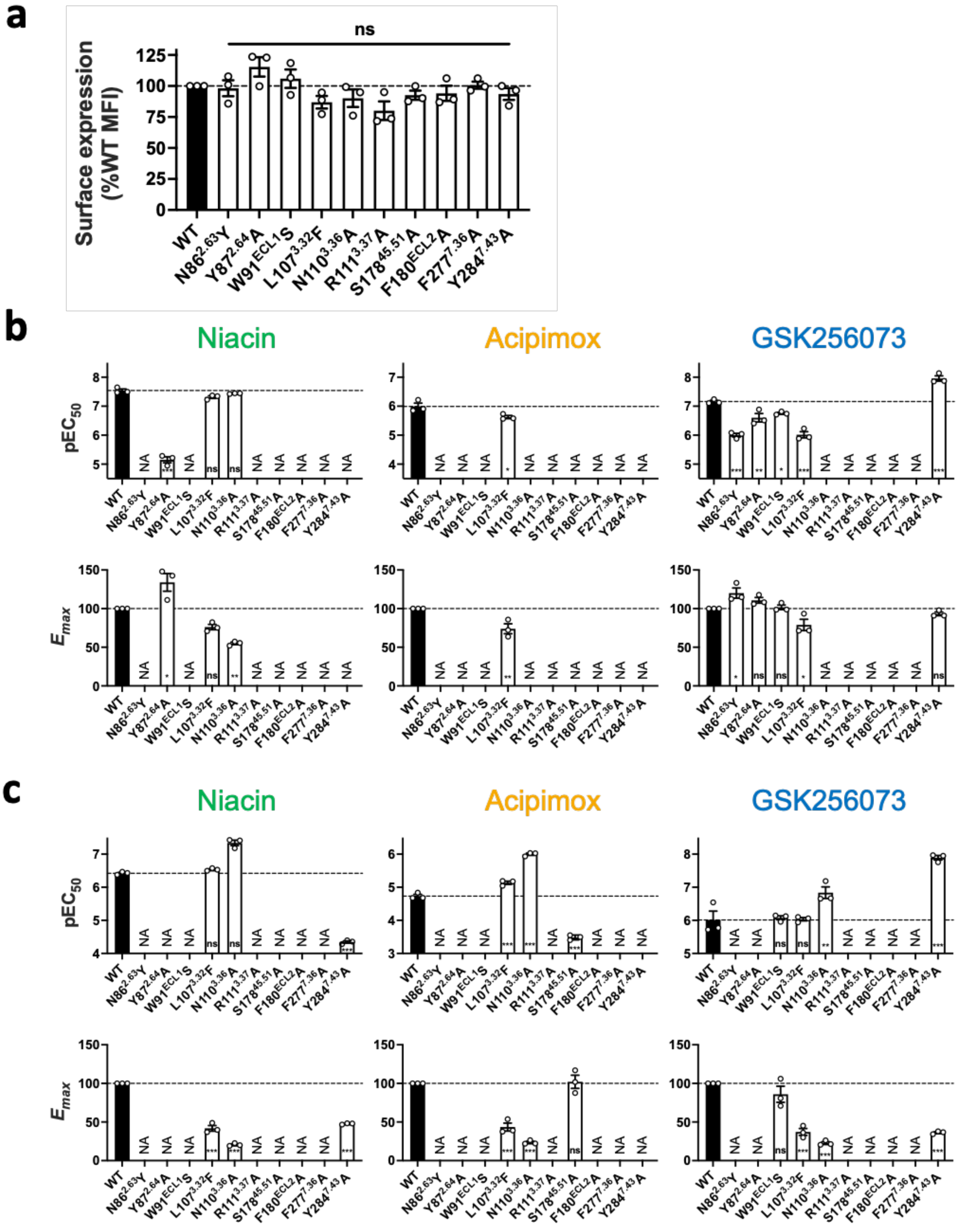

Supplementary Figure 6. Pharmacological parameters of of wild-type HCAR2 and mutants.

(a) Surface expression levels of the wild-type HCAR2 and mutants. MFI, mean fluorescent intensity. (b, c) Pharmacological parameters (pEC_{50}; negative logarithmic values of EC_{50} values, E_{max}; the maximum response) calculated from the NanoBiT assays for G_i dissociation. (b) and β-arrestin1 recruitment (c) upon stimulation with niacin, acipimox and GSK256073.

Bars and error bars represent mean and SEM, respectively, of three independent experiments with each data point represented as a dot. ns, not significantly different between the groups. For the statistical analyses, ** indicates $P < 0.01$ and *** indicates $P < 0.001$ with one-way ANOVA followed by the Dunnett's test for multiple comparison analysis (with reference to the wild-type HCAR2).

Supplementary Figure 7.

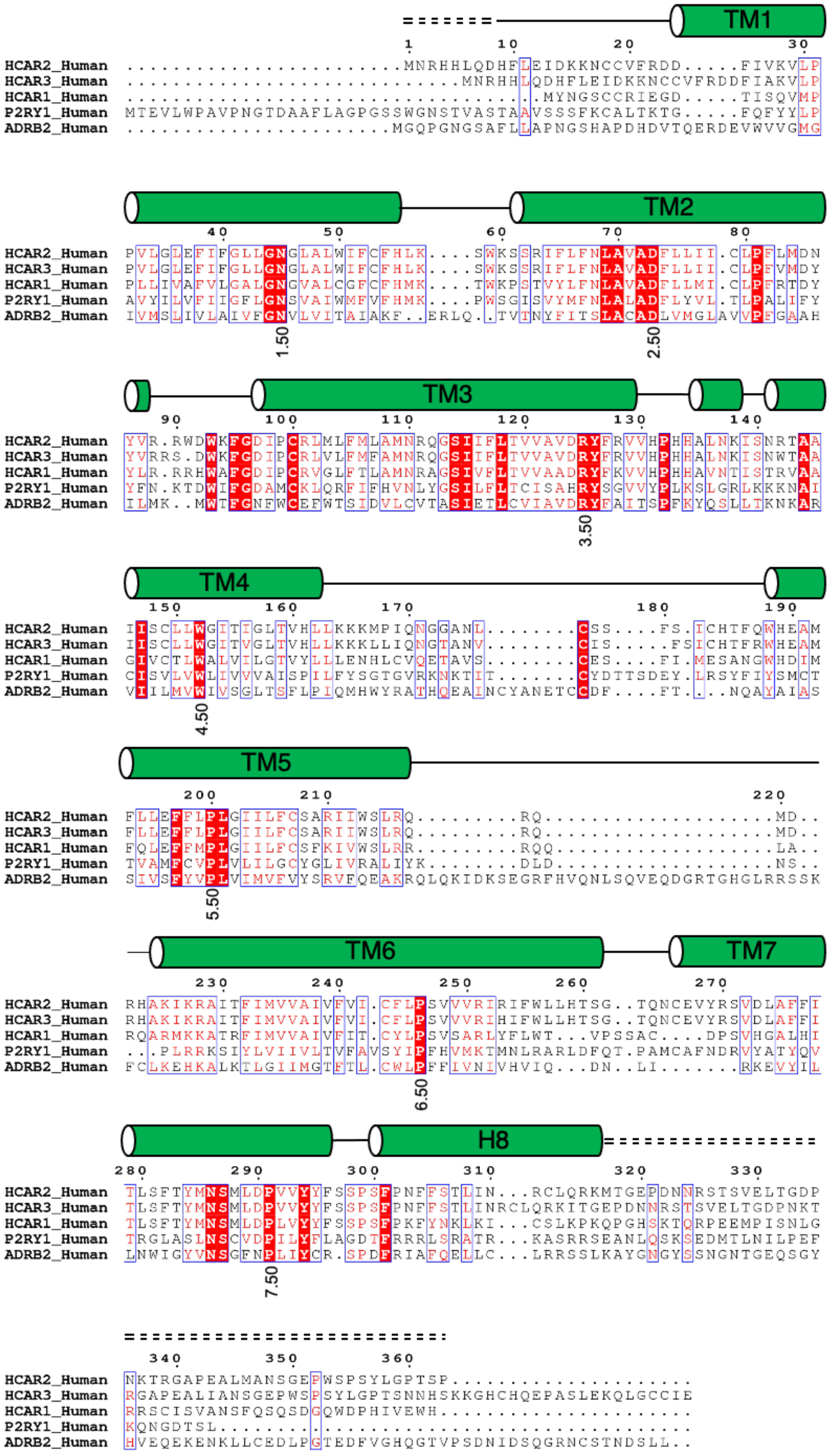

Supplementary Figure 7. Sequence alignment of the HCARs.

Sequence alignment of HCARs calculated with CLUSTALW. The bars mark the locations of helices in HCAR2. Identical residues are shown in red, and similar residues are boxed. The residues corresponding to Ballesteros-Weinstein numbers 1.50, 2.50, 3.50, 4.50, 5.50, 6.50 and 7.50 were labeled.

Supplementary Figure 8.

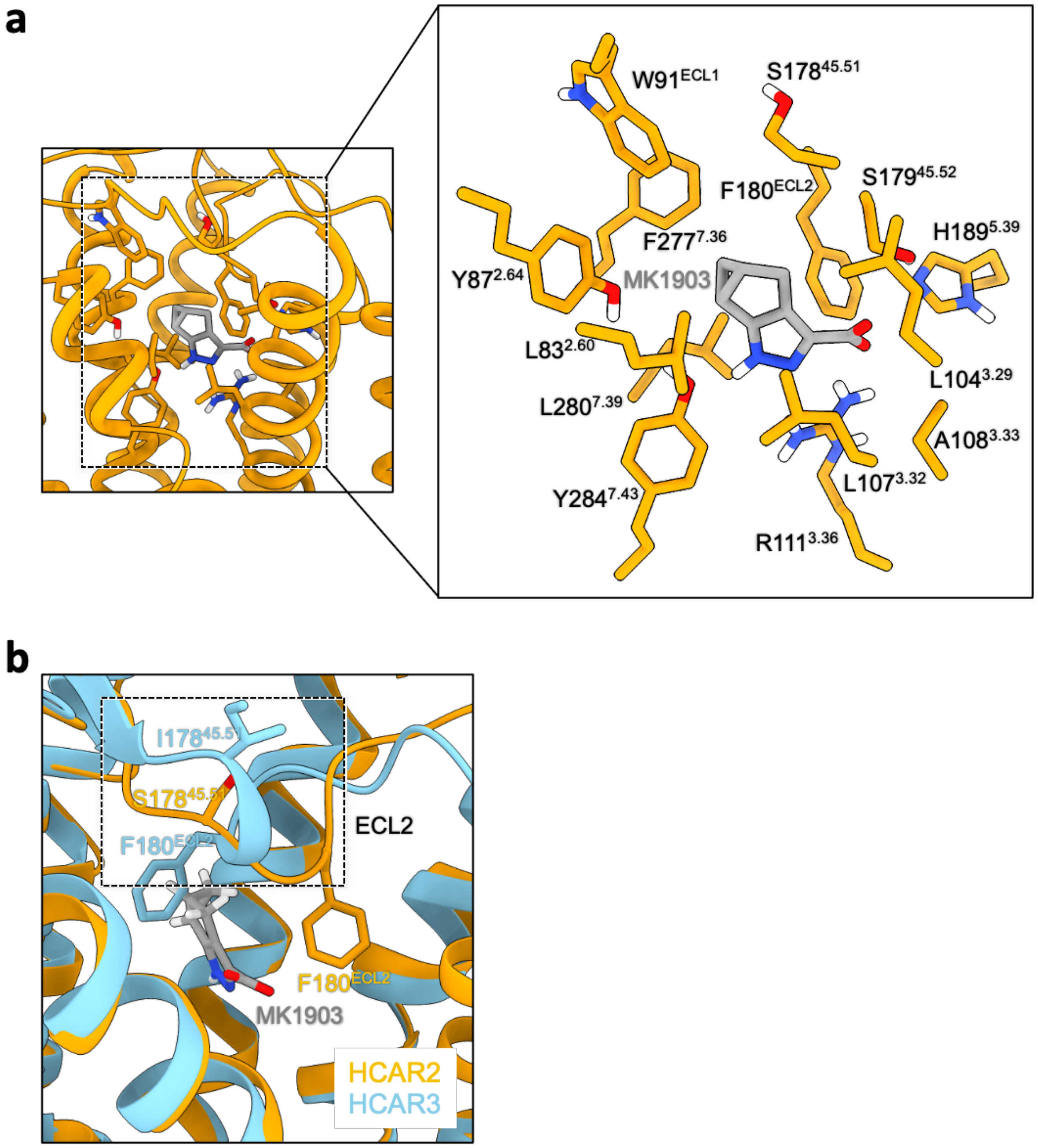

Supplementary Figure 8. Docking of MK-1903 on HCAR2 structure.
(a) Molecular docking model of MK-1903 on the acipimox bound HCAR2 structure (left). Magnified view of key residues interacting with MK1903 (right). (b) Magnified view of superimposed structure of the MK-1903 docked HCAR2 model on active HCAR3 model generated by Multi-state GPCR modeling based on AlphaFold2.

Supplementary Figure 9.

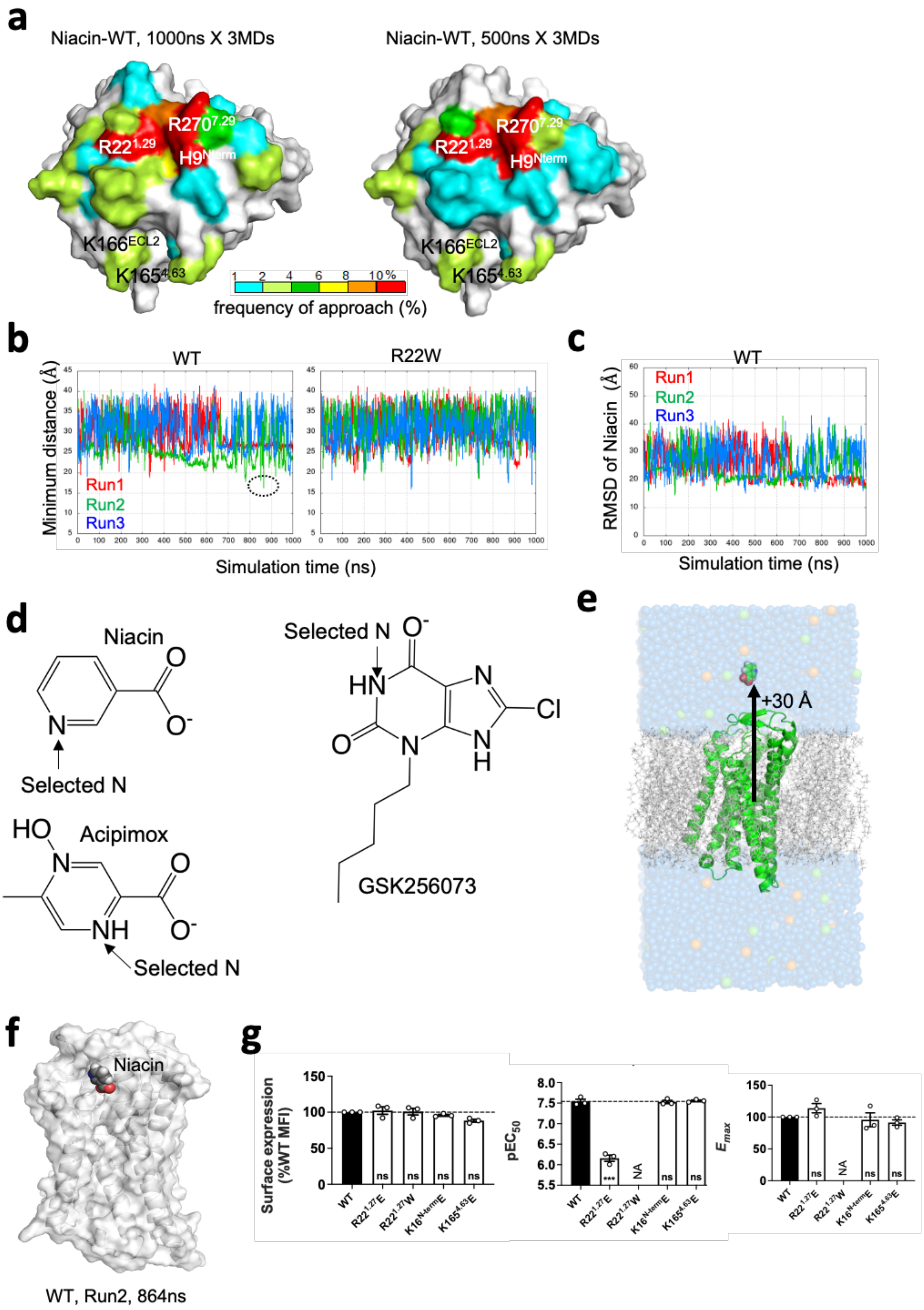

Supplementary Figure 9. MD simulations of ligand binding pathways and supporting experimental mutagenesis results.
(a) Residues of HCAR2 contacted by niacin, colored by frequency of contacts during 1000 ns MD (left) and 500 ns MD (right). (b) Time dependence of the minimum distance for the wild-type(left) and the R22$^{1.27}$W (right) mutant. (c) RMSD from the ligand pose at the orthosteric site: The structural alignment to the experimental structure model was done using Cα atoms for 9-299 residues of HCAR2 and RMSD was calculated using non-hydrogen atoms of niacin. (d) Ligand structures used in MD simulations and the selected nitrogen atom used in the z-direction flat-bottom restraint. (e) An MD unit cell of the membrane-water system of HCAR2 with niacin. (f) The snapshot of MD simulation of the wild-type HCAR2/niacin system with the lowest minimum distance. The niacin was represented as spheres. (g) Cell surface expression levels and pharmacological parameters of mutants related to ligand binding pathway.

Supplementary Figure 10.

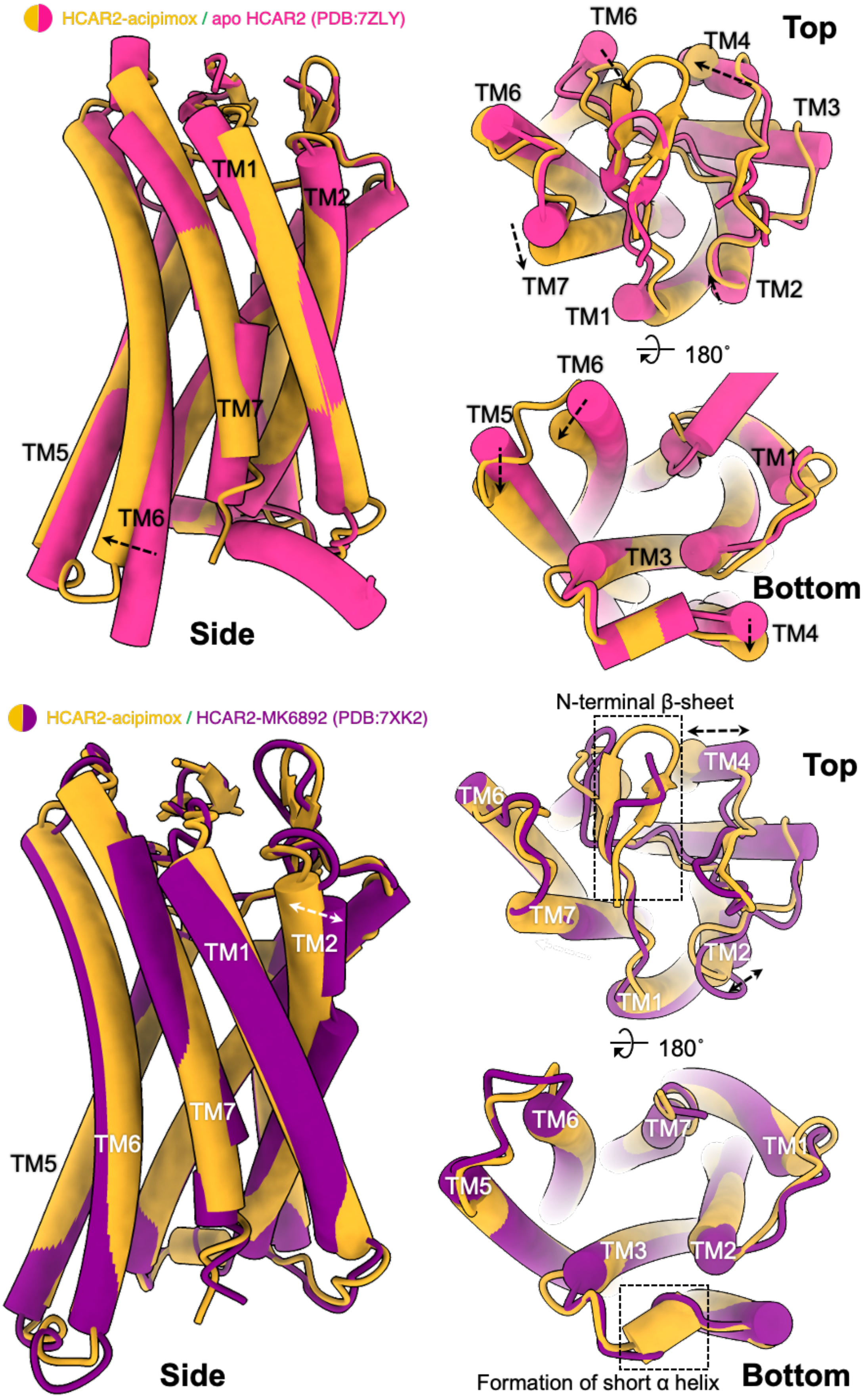

Supplementary Figure 10. Structural comparison of niacin, acpimox, GSK256073 and MK6892 bound HCAR2, with apo state inactive HCAR2.
Superimposition of the active niacin bound HCAR2 (green) and inactive P2Y1 model (grey). Side, top and bottom views of the overlaid structures are displayed using cylinders to represent helices. The structural movements upon receptor activation are indicated by dashed arrows. Formation of the short α helix on ICL2 is highlighted by a dashed box.

Supplementary Figure 11.

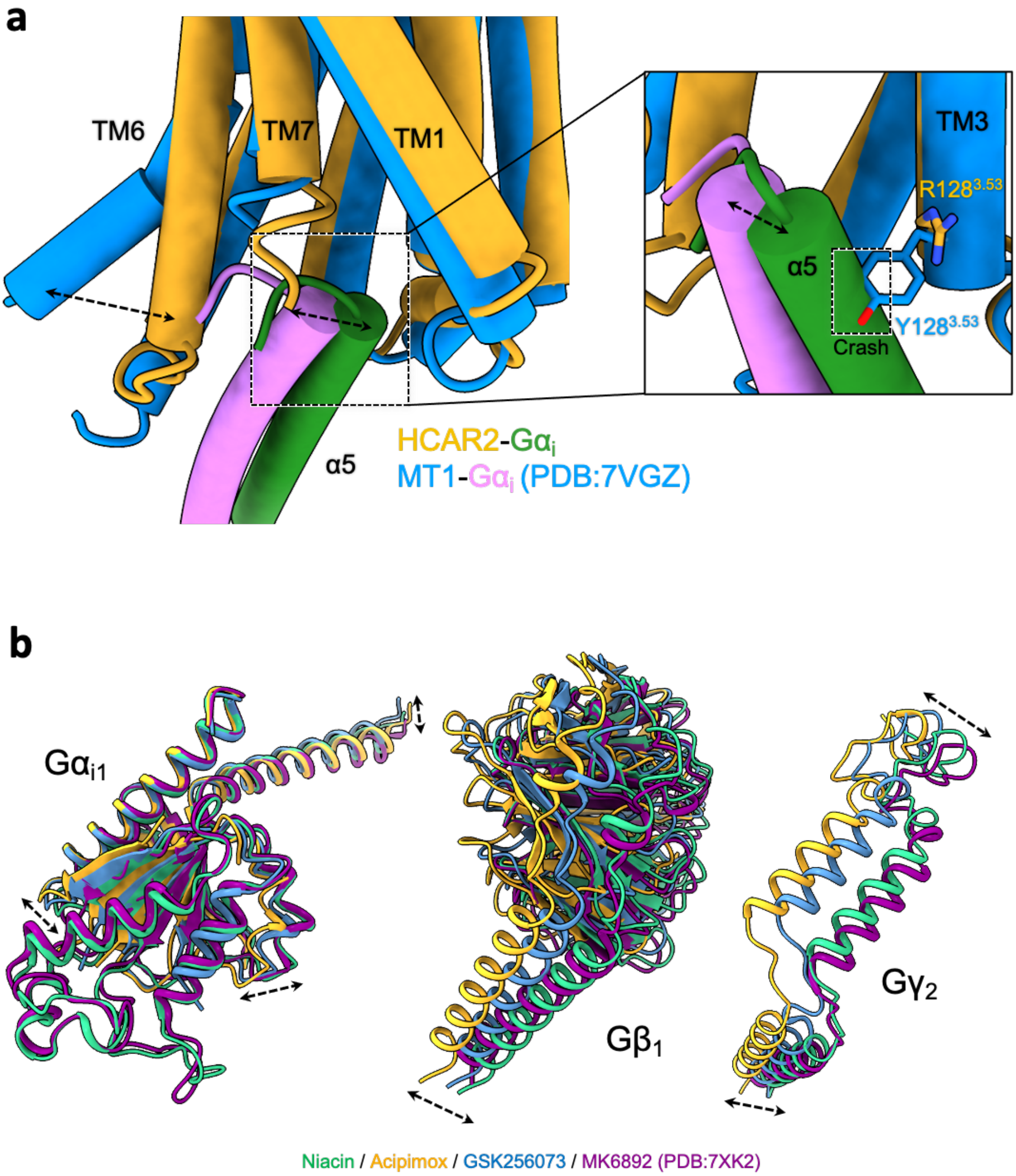

Supplementary Figure 11. Comparison of the G_i binding interface with the MT1-G_i complex and the movement of Gαβγ subunits.

(a) Superimposed structure of the acpimox bound HCAR2-G_i (orange) on Melatonin receptor type 1A (MT1)-G_i complex (PDB:7VGZ). Magnified view of G_i and receptor interface is shown on the right. (b) Gαβγ subunits of the niacin, acipimox, GSK256073, MK6892 (PDB:7XK2) bound complexes, aligned on receptor.

Supplementary Table 1. Statistics for data collection and structural refinement

	Niacin-HCAR2-G_i complex		Acipimox-HCAR2-G_i complex		GSK256073-HCAR2-G_i complex	
	Overall	Receptor (Local Refinement)	Overall	Receptor (Local Refinement)	Overall	Receptor (Local Refinement)
**PDB entry**	8H2G	8K5B	8I7V	8K5C	8I7W	8K5D
**EMDB entry**	EMD-34437	EMD-36900	EMD-35234	EMD-36901	EMD-35235	EMD-36902
**Data collection and processing**						
Magnification	105,000		105,000		105,000	
Microscope	Titan Krios G4		Titan Krios G4		Titan Krios G4	
Voltage (kV)	300		300		300	
Detector	Gatan K3 Summit		Gatan K3 Summit		Gatan K3 Summit	
Energy filter	Gatan Quantum-LS, 15 eV slit		Gatan Quantum-LS, 15 eV slit		Gatan Quantum-LS, 15 eV slit	
Electric exposure (e^-/Å)	51.7		51.7		51.7	
Deforcus range (µm)	-0.8 to -1.8		-0.8 to -1.8		-0.8 to -1.8	
Collection mode	CDS mode		CDS mode		CDS mode	
Pixel size (Å)	0.83		0.83		0.83	
Data Processing Program	cryoSPARC (v.4.0.3)		cryoSPARC (v.4.0.3) / Relion (v.4.0.0)		cryoSPARC (v.4.0.3) / Relion (v.4.0.0)	
Movies	5,001		4,301		4,957	
Initial / Final particle images (no.)	2,135,798 / 291,411		5,581,295 / 228,127		5,434,103 / 153,633	
Symmetry imposed	C1	C1	C1	C1	C1	C1
Map resolution (Å)	3.01	3.43	2.77	3.13	3.39	3.74
FSC threshold	0.143	0.143	0.143	0.143	0.143	0.143
**Refinement**						
Refinement Program			PHENIX (v.1.20.1)			
Model resolution (Å)	2.93	3.37	2.71	3.05	3.14	3.61
FSC threshold	0.143	0.143	0.143	0.143	0.143	0.143
Model composition						
Non-hydrogen atoms	9,023	2,374	8,374	2,380	8,461	2,345
Protein residues	1,145	291	1,066	291	1,080	291
R.m.s. deviations						
Bond length (Å)	0.003	0.004	0.004	0.004	0.003	0.003
Bond angles (°)	0.564	0.713	0.625	0.632	0.576	0.646
Validation						
MolProbuty score	1.76	2.04	1.84	1.74	1.83	1.97
Clashscore	9.49	12.54	9.49	8.34	11.87	11.34
Ramachandran plot						
Favored / Allowed (%)	96.20 / 3.80	94.46 / 5.54	97.14 / 2.77	95.85 / 4.15	96.42 / 3.58	94.12 / 5.88
Disallowed (%)	0.00	0.00	0.00	0.00	0.09	0.00
Mask CC	0.75	0.77	0.79	0.78	0.80	0.79

Supplementary Table 2. Truncated residues in HCAR2 models

Nterm	TM1	ICL1	TM2	ECL1	TM3	ICL2	TM4
H9	D23, D23,	S56	K60	K94	D97		
K15, K15	D23	K57, K57	P81		I98		
C19, C19	F25	S58, S58	M84		M106, M106		
	K28		D85		M109		

ECL2	TM5	ICL3	TM6	ECL3	TM7	H8
K166	F186		K225		C266, C266	P299
M167						

* Side-chain truncated residues in HCAR2 models are listed and colored as green (niacin bound), orange (acipimox bound) and blue (GSK256073 bound), respectively.

Supplementary Table 3. List of primers used to introduce HCAR2 mutants

HCAR2-K16E-Forward	GAGAACTGCTGTGTGTTCCGAGAT
HCAR2-K16E-Reverse	CTTGTCTATTTCCAGAAAGTG
HCAR2-R22E-Forward	GAGGATGACTTCATTGTCAAGGTG
HCAR2-R22W-Forward	TGGGATGACTTCATTGTCAAGGTG
HCAR2-R22-Reverse	GAACACACAGCAGTTCTTCTT
HCAR2-K165E_Forward	GAGAAGATGCCGATCCAGAATGGCGGT
HCAR2-K165-Reverse	CTTCAGGAGGTGGACTGTCAG
HCAR2-N86Y-Forward	CCCTTCCTGATGGACTACTATGTGAGGCGTTGG
HCAR2-N86Y-Reverse	CCAACGCCTCACATAGTAGTCCATCAGGAAGGG
HCAR2-Y87A-Forward	TTCCTGATGGACAACGCTGTGAGGCGTTGGGAC
HCAR2-Y87A-Reverse	GTCCCAACGCCTCACAGCGTTGTCCATCAGGAA
HCAR2-W91S-Forward	AACTATGTGAGGCGTAGCGACTGGAAGTTTGGG
HCAR2-W91S-Reverse	CCCAAACTTCCAGTCGCTACGCCTCACATAGTT
HCAR2-L107F-Forward	CTGATGCTCTTCATGTTTGCTATGAACCGCCAG
HCAR2-L107F-Reverse	CTGGCGGTTCATAGCAAACATGAAGAGCATCAG
HCAR2-N110A-Forward	TTCATGTTGGCTATGGCTCGCCAGGGCAGCATC
HCAR2-N110A-Reverse	GATGCTGCCCTGGCGAGCCATAGCCAACATGAA
HCAR2-R111A-Forward	ATGTTGGCTATGAACGCTCAGGGCAGCATCATC
HCAR2-R111A-Reverse	GATGATGCTGCCCTGAGCGTTCATAGCCAACAT
HCAR2-S178A-Forward	GGTGCAAATTTGTGCGCAAGCTTCAGCATCTGC
HCAR2-S178A-Reverse	GCAGATGCTGAAGCTTGCGCACAAATTTGCACC
HCAR2-F180A-Forward	AATTTGTGCAGCAGCGCAAGCATCTGCCATACC
HCAR2-F180A-Reverse	GGTATGGCAGATGCTTGCGCTGCTGCACAAATT
HCAR2-F277A-Forward	GTGGACCTGGCGTTCGCAATCACTCTCAGCTTC
HCAR2-F277A-Reverse	GAAGCTGAGAGTGATTGCGAACGCCAGGTCCAC
HCAR2-Y284A-Forward	ACTCTCAGCTTCACCGCAATGAACAGCATGCTGGA
HCAR2-Y284A-Reverse	TCCAGCATGCTGTTCATTGCGGTGAAGCTGAGAGT
