# Supplementary material for: Structural basis for ligand recognition and signaling of hydroxy-carboxylic acid receptor 2
Source: Nat Commun. 2023 Nov 6;14:7150. doi: 10.1038/s41467-023-42764-8 (PMC10628104; doi:10.1038/s41467-023-42764-8)
Supplement: Supplementary file 3 — Description of Additional Supplementary Files [file 41467_2023_42764_MOESM3_ESM.pdf]

Title: Supplementary Data 1.
Description: Pharmacological parameters of wild-type HCAR2 and mutants
Pharmacological parameters (pEC_{50}; negative logarithmic values of EC_{50} values, E_{max}; the maximum response) calculated from the NanoBiT assays for G_i dissociation. Mean and SEM of three independent experiments.
